# Impact of virtual education based on health belief model on cervical cancer screening behavior in middle‐aged women: A quasi‐experimental study

**DOI:** 10.1002/cnr2.2058

**Published:** 2024-04-10

**Authors:** Mahdieh Sadat Khoshnazar, Mohammad Javad Tarrahi, Hossein Shahnazi

**Affiliations:** ^1^ School of Health Isfahan University of Medical Sciences Isfahan Iran; ^2^ Department of Epidemiology and Biostatistics, School of Health Isfahan University of Medical Sciences Isfahan Iran; ^3^ Department of Health Education and Health Promotion, School of Health Isfahan University of Medical Sciences Isfahan Iran

**Keywords:** belief, cervical cancer, education, knowledge, pap smear

## Abstract

**Background:**

Cervical cancer is one of the most common cancers in women worldwide and a cause of high mortality among people. Pap smear screening is an appropriate method to prevent cervical cancer and reduce its mortality.

**Aim:**

This study aimed to determine the effect of web‐based education based on the Health Belief Model (HBM) on cervical cancer screening behavior in middle‐aged women.

**Methods and Results:**

This study is a quasi‐experimental interventional research that was conducted on 240 middle‐aged women aged 40–59 years in Isfahan, Iran, in 2022. An online educational intervention based on the constructs of the Health Belief Model was conducted for the intervention group using the Triple‐B platform. The information on the intervention and control groups was collected before, immediately after the intervention, and 2 months later using a valid questionnaire. The gathered Data was analyzed using ANOVA and LSD post‐hoc, independent samples *t* test, chi‐square, and MANCOVA statistical tests in SPSS 26 software. After the intervention, the mean scores of knowledge, perceived susceptibility, perceived severity, perceived benefits, self‐efficacy, and internal cues to action in the intervention group increased and the mean score of perceived barriers decreased (*p* < .001). The mean score of the external cues to action did not show a significant difference between the intervention and control groups before, immediately, and 2 months after the intervention. Two months after the intervention, 32 women (26.2%) in the intervention group and two women (1.7%) in the control group performed the Pap smear test.

**Conclusion:**

Web‐based educational intervention based on HBM using different strategies such as question and answer, presentation of infographics, lectures, brainstorming, showing videos and numerous educational images can be an effective way for increasing knowledge and cognitive variables of women and doing Pap smear test.

## 1 INTRODUCTION

Cervical cancer is one of the most common cancers among women worldwide. In 2020, the number of new cases of this cancer was estimated to be 604 000, and the number of deaths due to this disease was 342 000 around the world.^1^ Low‐ to middle‐income countries account for about 90% of deaths caused by this cancer.^2^ According to the World Health Organization's prediction, the number of deaths due to cervical cancer will reach about 474 000 per year by 2030, and 95% of these deaths will be related to low‐ and middle‐income countries.^3^ The incidence of this disease in Iran has an upward trend^4^ and due to the late diagnosis of the disease, the mortality rate is also high.^5^

Cervical cancer is recognized as a preventable disease due to the existence of effective primary and secondary prevention programs, namely vaccination against the main causative agent, human papillomavirus (HPV), and early detection programs.^1^ Prevention and early diagnosis of cervical cancer are critical factors in reducing the incidence and mortality of this disease.^6^ Preventive measures are not uniformly implemented across countries. In some countries, including Iran, women have not been vaccinated against HPV, which highlights the heightened importance of early detection programs in such cases.^1^ Studies have indicated that the 5‐year survival rate for patients in Iran stands at 58%,^7^ whereas in countries such as Sweden and Taiwan, it reaches 70%.^8^, ^9^ Since this cancer initially undergoes a long pre‐invasive period (about 20 years), early detection of the disease at this time and treatment of the initial lesions can prevent abnormal growth of lesions to a considerable extent.^3^ In this regard, an effective measure to detect lesions in the pre‐invasive stage and reduce mortality due to this disease is screening.^10^ Generally, when the disease is diagnosed in its early stages through regular screening, the 5‐year survival rate of the patient is 92%, but when the disease is advanced, this rate drops to about 13%.^11^ One of the most effective methods of screening for cervical cancer is the Pap smear test, which can significantly reduce mortality and incidence of the disease.^12^ This test is a simple, inexpensive, and harmless method^13^ that creates an opportunity for timely treatment by early detection of abnormal cells during the pre‐invasive stage and prevents the incidence of disease.^1^ Effective measures such as HPV vaccination and Pap smear screening in Nordic countries such as Denmark, Sweden, and Finland, which planned and implemented extensive screening programs for the early detection of cervical cancer from the early 1950s, have resulted in a significant reduction of 10%–80% in mortality rates caused by cervical cancer in these countries.^14^ Evidence shows that the implementation of the Pap smear screening program can reduce the incidence of cervical cancer by 79% and the mortality from this disease by 70%.^10^

According to the guidelines of the Ministry of Health of Iran, all women are required to undergo a Pap smear test after marriage, and after having three reliable negative tests over three consecutive years, this screening should be repeated once every 3 years.^15^ However, studies show that only 3%–14.8% of eligible individuals in Iran have regularly undergone Pap smear testing.^5^, ^15^

Researches conducted in different regions of the world indicate that women do not participate in Pap smear screening programs for various reasons. In many of these studies, lack of knowledge about cervical cancer and Pap smear screening and wrong beliefs in this regard are mentioned.^16^, ^17^, ^18^ In other studies, In addition to poor knowledge and incorrect beliefs, low perception of susceptibility to cancer has also been introduced as a reason for not undergoing Pap smear testing in women.^12^, ^19^ Furthermore, barriers such as fear of pain during the test and misconceptions about the test,^20^ fear of test results, the feeling of shame and embarrassment of undergoing the screening, as well as lack of understanding of the importance and benefits of screening, have been considered as important reasons for not undergoing screening.^21^ Many studies conducted on cervical cancer screening behaviors have examined only one or a few influencing factors. In this study, a set of cognitive factors was investigated and intervened in the form of the Health Belief Model (HBM).

HBM specifically focuses on the adoption of preventive health behaviors^22^ and emphasizes that the adoption of these behaviors is dependent on individual perceptions and beliefs regarding the fear of illness or health problems, as well as understanding and evaluating the benefits and barriers to the desired behavior.^6^, ^23^ According to HBM, if individuals perceive the risk and susceptibility to a disease such as cervical cancer, the severity of that disease and its consequences, and the benefits of desired preventive behavior, and feel fewer barriers to performing the correct behavior and have enough confidence in their ability to overcome those barriers, they will adopt preventive behavior. In addition, having cues to action that trigger people to change behavior can help them to do screening behavior.^24^

### 1.1 Objectives of the research

The present study aimed to determine the effect of web‐based education based on the HBM regarding cervical cancer screening behavior in middle‐aged women.

Research hypothesis: This study is conducted to survey the following hypothesis:

1. The mean scores of the constructs of HBM increase significantly after the implementation of the educational intervention in the intervention group.
2. The frequency of performing Pap smear tests increases significantly after the educational intervention in the intervention group.

## 2 METHODS

### 2.1 Study design and participants

This study is a quasi‐experimental interventional research that was conducted in 2022 on middle‐aged women in Isfahan, Iran. The statistical population included all middle‐aged women covered by health centers in Isfahan city. Inclusion criteria included: being married at least once (including married, widowed, and divorced women), age 40–59, not having a Pap smear test in the past 5 years, having no history of hysterectomy, cervical cancer, or other cancers, not being pregnant, ability to use WhatsApp and also participate in web‐based sessions, and willingness to participate in the study. Failure to complete each of the pre‐test and post‐test questionnaires, absence from at least one training session, and unwillingness to continue cooperation in the study were considered exclusion criteria.

### 2.2 Sample size and sampling

The sample size was calculated according to the following formula:

$$n=\frac{{\left(Z_{1-\frac{\propto }{2}}+Z_{1-\beta }\right)}^{2}\times 2\sigma^{2}}{d^{2}}.$$

The values of $z_{1-\frac{\alpha }{2}}$ and $z_{1-\beta }$ are equal to 1.96 and 0.84, respectively.^4^ The standard deviation and the difference between the means of the intervention and control group are equal to σ=12 and *d* = 5, respectively. It should be noted that the considered variable is behavior.^4^

Accordingly, the sample size for each group was calculated as 91. Considering an attrition rate of 30% in each group, the final sample size was determined to be 120 in each group.

**FIGURE 1:**
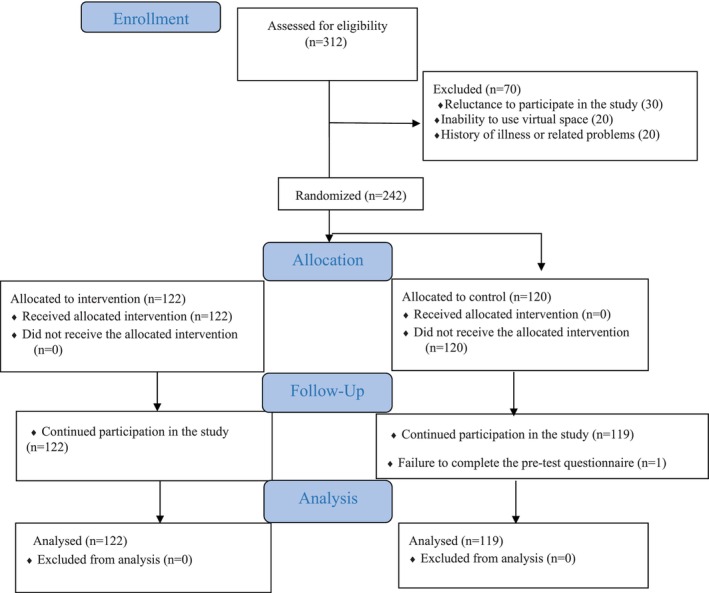
CONSORT flow diagram (Trial Profile).

The sampling method was multistage random. In this way, among the two health centers No. 1 and 2 in Isfahan City, center No. 2 was randomly selected. Then, four sub‐centers (called comprehensive health centers) were randomly selected. By referring to four selected health centers, the list of women eligible to participate in the study was collected through the comprehensive health information system called SIB. Then, we made phone calls to selected individuals and fully explained the objectives and conditions of the study to them. Those who had consent to participate in the study were selected as the final sample. These calls continued until 60 women were selected from each center. Finally, 30 women were assigned to the intervention and 30 women to the control group using Random Allocation software version 1.0.0 (Figure 1).

### 2.3 Measurements

Information was collected using the questionnaire of a similar study.^25^ The study questionnaire consisted of three sections. The first section included demographic questions (19 questions), the second section was knowledge questions (23 questions), and the third section was related to the constructs of HBM which included perceived susceptibility (six questions), perceived severity (five questions), perceived benefits (five questions), perceived barriers (14 questions), perceived self‐efficacy (five questions), external and internal cues to action (11 questions each), and behavior (one question). The questions were scored as follows: For the knowledge section, a correct answer received 2 points, an incorrect answer received 0 points, and “I don't know” received 1 point. A 5‐point Likert scale was used for the questions of HBM constructs, and the score range of each question varied from 1 to 5. In the behavior section, the women asked about performing the pap smear test and related documents in this regard requested from them. This questionnaire was provided to both intervention and control groups via WhatsApp before, immediately after the intervention, and 2 months later.

### 2.4 Validity and reliability of the questionnaire

Content validity was used to assess the validity of the tool. The questionnaire was offered to nine academic members of health education and midwifery, and their suggested modifications were made so that, the questionnaire's validity was confirmed.

To check the reliability, the questionnaire was completed by 20 individuals who were similar to the study population in terms of demographic variables. For the knowledge questions the split‐half method was used and for HBM constructs Cronbach's alpha coefficient was used. The Spearman‐Brown coefficient for knowledge questions was 0.77 and Cronbach's alpha for perceived susceptibility, perceived severity, perceived benefits, perceived barriers, and perceived self‐efficacy was obtained at 0.71, 0.71, 0.75, 0.85, and 0.86 respectively.^25^

### 2.5 Intervention

The educational intervention based on HBM constructs was held for 3 weeks in five 1‐h web‐based training sessions using the Triple‐B platform for the intervention group. The first session was held to increase knowledge about cervical cancer and Pap smear screening and increase women's perceived susceptibility. In this session, an overview of cancers with an emphasis on cervical cancer, information about Pap smear testing, and the ways to prevent the disease, symptoms, and risk factors were presented through lectures, slide presentations, infographics, question and answer, and discussions with participants. In the second session, the emphasis was on understanding the severity of the disease and the seriousness of the complications, consequences, and problems caused by it. This was accomplished by utilizing slide presentations and images, as well as engaging individuals in discussions to provide examples of relevant observations. The third session, aimed to increase the perceived benefits and decrease the perceived barriers to undergoing the Pap smear test. In this session, a midwife was invited to provide training regarding the pap smear test, the benefits and process of performing a Pap smear test, correcting any misunderstandings in this regard, and removing the perceived barriers. Multiple slides and images were utilized to reinforce the presented material. Finally, different scenarios were used to encourage active participation in the discussion. In the fourth session, to enhance women's self‐efficacy about performing Pap smear screening, we provided training on the skills of overcoming fear and improving self‐confidence through slide presentations, lectures, and question and answer. In addition, we tried to present objective patterns by involving some audience members in the discussion who had been able to overcome the existing barriers in the past and perform the pap smear test. We also used short videos in this regard. The fifth session was also about increasing women's knowledge of internal and external cues to action, which was done through brainstorming techniques, slide presentations, and lectures. Women were acquainted with warning signs of diseases as well as reliable sources of information, such as midwifery journals, health‐related TV channels, trustworthy websites, books, and how to access them. Finally, after conducting two stages of post‐test, a copy of the screening result was received from individuals who underwent the Pap smear test after the training and was attached to the questionnaires. Also, during these 2 months, reminder messages in the form of images, text, pamphlets, and short videos were sent to the intervention group members. It should be noted that at the end of the program, after completing the final stage, the entire educational content was made available to the control group so they could also benefit from the information and training.

### 2.6 Ethical considerations

This study has been approved by the Research Ethics Committee of Isfahan University of Medical Sciences with the ethical code of IR.MUI.RESEARCH.REC.1400.543. Participants were assured of the confidentiality of their information. Participation in the study was voluntary and individuals could withdraw from the research at any stage. After informing the participants about these issues, as well as the goals and methods of the study, their written consent was also obtained.

### 2.7 Data analysis

SPSS‐26 was used for data analysis. First, the normality of the data was confirmed using the Kolmogorov–Smirnov test. Then, independent samples *t* test and chi‐square test were used to compare demographic variables between the two groups. In addition, to evaluate the mean scores of HBM constructs and knowledge at different time points, the repeated measures ANOVA and LSD post‐hoc tests were used. Also, to compare the two intervention and control groups in terms of the trend of changes in the score of knowledge and model constructs during the study, a repeated measures ANOVA test was used. To compare the difference between the intervention and control groups, in terms of mean scores of HBM constructs and knowledge, in the pre‐intervention stage, independent samples *t* test, and in each of the post‐intervention stages MANCOVA test was used. The chi‐square test was used to compare behavior (performing or not performing Pap smear) between the two groups after the intervention. The significance level was considered less than 0.05.

## 3 RESULTS

This study was conducted on 122 women in the intervention group and 119 women in the control group. Independent samples *t* test showed that the mean and standard deviation of participants' age in the intervention and control groups were 44.42 ± 4.44 and 46.85 ± 5.65 years respectively. So, there was a significant difference between the two groups in terms of age (*p* < .001), which was adjusted as a confounding variable in the data analysis process. There was no statistically significant difference between the two groups in terms of other demographic variables (Tables 1, 2).

**TABLE 1:** Comparison of mean and standard deviation of demographic variables between intervention and control groups.

Variable	Intervention Group	Control Group	*p* value^a^
	Mean ± SD	Mean ± SD	
Age	44.42 ± 4.44	46.85 ± 5.65	<.001
Marriage age	23.20 ± 4.74	23.88 ± 4.16	.23
Age at first pregnancy	25.53 ± 4.80	26.09 ± 4.69	.37
gravidity	2.35 ± 1.12	2.10 ± 1.10	.08

^a^ Independent samples *t* test.

**TABLE 2:** Comparison of frequency of demographic variables between intervention and control groups.

Variable		Intervention group	Control group	*p* value^a^
		*N* (%)	*N* (%)	
Marital status	Married	119 (97.50)	112 (94.12)	.18
	Divorced or widowed	3 (2.50)	7 (5.88)	
Occupation	Housewife	76 (62.29)	68 (57.14)	.41
	Employed	46 (37.71)	51 (42.86)	
Education	Under diploma	11 (9)	10 (8.40)	.55
	Diploma	61 (50)	52 (43.70)	
	University	50 (41)	57 (47.90)	
Smoking or hookah use	Yes, smoking	5 (4.10)	2 (1.68)	.47
	Yes, hookah	8 (6.56)	10 (8.40)	
	No	109 (89.34)	107 (89.92)	
History of using contraceptive pills	Yes	59 (48.36)	67 (56.30)	.21
	No	63 (51.64)	52 (43.70)	

^a^ Chi‐square test.

Before the intervention, there was no statistically significant difference between the two groups in knowledge and HBM constructs (Table 3). Immediately and 2 months after the intervention, there was a significant difference between the intervention and control groups in terms of the mean score of knowledge and the constructs of perceived susceptibility, perceived severity, perceived benefits, perceived barriers, self‐efficacy, and internal cues to action (Table 3). The calculation of intervention p‐value for knowledge and constructs of the model showed that there was a significant difference between the intervention and control groups in terms of the trend of changes in scores of knowledge, perceived susceptibility, perceived severity, perceived benefits, perceived barriers, self‐efficacy, and internal cues to action. In this way, this trend of changes was constant for the control group during the study, while for the intervention group, it showed a decreasing trend for perceived barriers and an increasing trend for knowledge and other constructs. Additionally, there was no significant difference in the trend of changes in scores for external cues to action between the two groups, and this trend was constant for both groups (Table 3).

**TABLE 3:** Comparison of knowledge and Health Belief Model (HBM) constructs in two groups before, immediately and 2 months after the intervention.

	Time	Intervention group	Control group	*p* value
		SD ± mean	SD ± mean	
Knowledge	Before the intervention	31.21 ± 5.71	31.15 ± 4.83	.92**
	Immediately after the intervention	39.008 ± 5.31	31.13 ± 4.92	<.001***
	Two months after the intervention	40.31 ± 3.73	31.02 ± 4.85	<.001***
	*p* value (time)*	<.001	.76	–
	*p* value (intervention)*	<.001		–
Perceived susceptibility	Before the intervention	18.68 ± 4.11	18.33 ± 3.36	.46**
	Immediately after the intervention	21.15 ± 3.11	18.57 ± 3.42	<.001***
	Two months after the intervention	21.27 ± 2.9	18.51 ± 3.48	<.001***
	*p* value (time)*	<.001	.24	–
	*p* value (intervention)*	<.001		–
Perceived severity	Before the intervention	18.91 ± 3.20	19.09 ± 2.88	.65**
	Immediately after the intervention	20.64 ± 2.61	19.22 ± 2.77	<.001***
	Two months after the intervention	21.10 ± 2.27	19.23 ± 2.73	<.001***
	*p* value (time)*	<.001	.42	–
	*p* value (intervention)*	.003		–
Perceived benefits	Before the intervention	21.48 ± 2.61	21.14 ± 2.37	.29**
	Immediately after the intervention	23.44 ± 1.33	21.01 ± 2.26	<.001
				***
	Two months after the intervention	23.63 ± 1.20	20.96 ± 2.07	<.001***
	*p* value (time)*	<.001	.23	–
	*p* value (intervention)*	<.001		–
Perceived barriers	Before the intervention	31.07 ± 8.06	32.71 ± 7.04	.09**
	Immediately after the intervention	28.15 ± 6.74	32.52 ± 6.97	<.001***
	Two months after the intervention	27.95 ± 6.44	32.63 ± 6.66	<.001***
	*p* value (time)*	<.001	.68	–
	*p* value (intervention)*	<.001		–
Perceived self‐efficacy	Before the intervention	19.4 ± 3.94	18.42 ± 4.43	.07**
	Immediately after the intervention	20.53 ± 3.49	18.38 ± 4.28	<.001***
	Two months after the intervention	20.44 ± 3.15	18.26 ± 3.94	<.001***
	*p* value (time)*	<.001	.57	–
	*p* value (intervention)*	<.001		–
Internal cues to action	Before the intervention	34.49 ± 11.07	34.40 ± 9.37	.94**
	Immediately after the intervention	40.52 ± 8.18	34.35 ± 8.89	<.001***
	Two months after the intervention	42.19 ± 6.78	34.26 ± 8.76	<.001***
	*p* value (time)*	<.001	.82	–
	*p* value (intervention)*	<.001		–
External cues to action	Before the intervention	30.17 ± 7.67	30.43 ± 7.79	.79**
	Immediately after the intervention	30.61 ± 6.67	30.73 ± 7.56	.94***
	Two months after the intervention	31.09 ± 6.14	30.42 ± 7.51	.07***
	*p* value (time)*	.16	.407	–
	*p* value (intervention)*	.665		–

* Repeated measures ANOVA.

** Independent samples *t* test.

*** MANCOVA.

In the control group, the mean score of knowledge and HBM constructs did not change significantly during the study period. However, in the intervention group, the mean scores of knowledge, perceived susceptibility, perceived severity, perceived benefits, self‐efficacy, and internal cues to action significantly increased immediately and 2 months after the intervention, and the mean score of perceived barriers decreased (Table 3). LSD post‐hoc tests showed that 2 months after the intervention, the mean scores of knowledge, perceived severity, perceived benefits, and internal cues to action in the intervention group were significantly higher than immediately after the intervention (*p* < .001). However, regarding the mean score of perceived susceptibility (*p* = .55), self‐efficacy (*p* = .56), and perceived barriers (*p* = .35), no significant differences were observed between immediate and 2 months after the intervention.

The mean score of the external cues to action did not show a significant difference between the intervention and control groups before, immediately, and 2 months after the intervention. During the study, the mean score of external cues to action did not change significantly in either of the intervention and control groups (Table 3).

Before the intervention, none of the individuals in the intervention and control groups had undergone a Pap smear test in the past 5 years however, 2 months after the intervention, the frequency of Pap smear testing in the intervention group was significantly higher than this frequency in the control group (Table 4).

**TABLE 4:** Comparison of the frequency of Pap smear tests 2 months after the intervention between the intervention and control groups.

Variable	Group	|Before the intervention	Two months after the intervention *N* (%)
Undergoing Pap smear test	Intervention	0	32 (26.22)
	Control	0	2 (1.68)
*p* value^a^			<.001

^a^ Chi‐square test.

## 4 DISCUSSION

The present study aimed to determine the effect of web‐based education based on HBM on the behavior of cervical cancer screening in middle‐aged women. The results showed that the web‐based educational program significantly improved participants' knowledge, perceived susceptibility, perceived severity, perceived benefits, perceived barriers, perceived self‐efficacy, and internal cues to action, which led them to perform a Pap test.

The intervention resulted in a significant increase in knowledge scores within the intervention group. The results of a study in the same field that was conducted using face‐to‐face training and group discussion and brainstorming techniques showed that the educational intervention has increased the mean knowledge score.^26^ In another study, where the content based on HBM constructs was delivered to the intervention group via Telegram instant messaging service, similar results were observed.^27^ In a similar study, conducted with women referring to healthcare centers in Bandar Abbas (southern Iran), after 3 months of intervention, the intervention group (consisting of 80 participants) who received educational posts on cervical cancer screening via WhatsApp showed a significant increase in their knowledge scores.^28^ Furthermore, the results of a review study based on the HBM are consistent with the findings of the current study.^10^

In addition to utilizing lecture techniques, this study promoted deep thinking among the audience regarding cervical cancer through techniques such as question and answer. The use of methods such as providing infographics not only made education more attractive but also facilitated learning, resulting in an improvement of knowledge in the intervention group.

Furthermore, the intervention resulted in a significant increase in perceived susceptibility scores and perceived severity scores in the intervention group and indicates the effectiveness of education in creating mental belief regarding the susceptibility to cancer, the severity and seriousness of the complications, and the consequences of cervical cancer. The results of a theoretical‐based study conducted on a group of women in the city of Fasa (Iran), focusing on Pap smears, utilizing face‐to‐face training and applying group discussion techniques, brainstorming, question and answer, video presentations, and PowerPoint, are consistent with the findings of the current study.^13^ In another study, which was conducted in Iran, three face‐to‐face training sessions based on the constructs of the Health Belief Model were implemented for 80 rural women, and similar results were reported.^11^ Also, the results of other previous studies are consistent with these results.^10^, ^26^, ^27^ The current educational intervention using techniques such as lectures and question and answer led to an increase in individuals' perception of the risk of disease. Furthermore, by sharing images and videos as well as engaging the audience in discussions to express their external observations on a web‐based platform, the complications and problems caused by the disease, the costs, and the difficulty of the treatment were concretely demonstrated to individuals. Therefore, this study demonstrates that the implementation of appropriate instructional techniques, even in the context of virtual classes and distance learning, can increase perceived susceptibility and severity, as same as to face‐to‐face education. So, in the current study, the implementation of online distance learning via the Triple‐B platform for educating on the prevention of cervical cancer is one of the novelties compared to other mentioned studies.

In addition, the intervention led to a significant increase in perceived benefits and a significant decrease in perceived barriers in the intervention group. Also, a similar study based on the Health Belief Model conducted through Telegram^27^ and the other previous studies confirm the current findings.^10^, ^11^, ^13^, ^26^ In fact, the present study demonstrated the importance and benefits of Pap smear testing and removed mental barriers by employing a midwife and utilizing her lecture and teachings. Additionally, by presenting numerous images, ambiguities regarding the tool and process of testing, as well as some other barriers, were resolved. The most important technique that improved these constructs was presenting scenarios and encouraging individuals to participate in online discussions. Therefore, since synchronous online classes in a virtual platform like Triple‐B have the capability of facilitating mutual interactions, are completely different from other virtual methods such as Telegram that have been used in previous studies. On the other hand, this virtual education overcomes time and location constraints compared to in‐person training. Thus, it can be used to correct people's perceptions of the benefits and barriers of screening.

In the current study, the self‐efficacy construct increased significantly after the education in the intervention group. The result of the same study showed that implementing educational intervention using a combination of offline multimedia messages and online class result in improving self‐efficacy, in terms of doing Pap smear.^3^ Also, other studies based on HBM showed that educational interventions can be effective in this regard.^10^, ^11^ The most important action that led to the change in self‐efficacy in the present study, was providing objective patterns through role model individuals who had succeeded in overcoming their fears and doing pap smears, as well as engaging the audience in discussion and using their previous experiences. In addition, some skills to overcome the fears were presented to the participants. Although, improving self‐efficacy seems difficult and requires skills training and extensive communication and interaction with individuals, which is usually addressed in face‐to‐face interventions, this study effectively managed to enhance the self‐efficacy of women regarding Pap smear through appropriate and effective techniques.

In this study, applying strategies such as brainstorming and online lectures led to an increase in the internal cues to action in the intervention group and promoted learning and retention of the contents in the audience's mind. By external cues to action, we mean credible sources of information that individuals can use to obtain accurate information on cervical cancer. The lack of influence of education on this construct, is likely because that more time is needed to follow up people in this sense, and in 2 months, they may not be exposed to such resources. In an intervention study that utilized face‐to‐face group education, the mean score of cues to action significantly increased after the intervention.^26^ However, this construct has not been reported in most studies.^13^, ^27^

Two months after the intervention, 32 individuals (26.22%) in the intervention group underwent a Pap smear test. The results showed that the educational intervention affected behavior; however, it is expected that a longer follow‐up period would result in a higher percentage of individuals in the intervention group undergoing the test. The findings of this study are consistent with studies that have found educational intervention to be effective in increasing Pap smear tests.^3^, ^10^, ^27^, ^28^ But the results of the intervention conducted through multimedia touch screen kiosks contradicted the findings of the present study and showed that the educational intervention had no impact on behavior. This could be due to the fact that the study only used individual and self‐paced learning methods, ignoring interpersonal interactions in education.^29^

The overall findings of this study were in line with several previous studies.^11^, ^27^, ^30^, ^31^ although there was a notable distinction in the methodology. While most prior research relied on face‐to‐face or messaging‐based training using platforms like Telegram or WhatsApp, this study employed web‐based training within a virtual platform, which demonstrated remarkable effectiveness. Virtual training on the Triple‐B platform offers several key advantages, including reduced time and cost requirements, as well as simplified implementation compared to in‐person approaches. Furthermore, this virtual education, compared to other virtual methods such as training via WhatsApp and Telegram, has the capability of establishing mutual interactions and employing interactive strategies such as group discussions.

### 4.1 Strengths

One of the main strengths of this study is the utilization of virtual education on the Triple‐B platform. This approach effectively eliminates the time and location limitations commonly associated with traditional face‐to‐face education. Additionally, this platform allows for the remote implementation of a wide range of interactive strategies. Simultaneously training a large number of participants online can be efficient and cost‐effective. It allows for a diverse range of individuals to be included, which can enhance the generalizability of the findings. Additionally, after the intervention, the behavior was assessed not only through self‐report but also objectively by obtaining a version of the individuals' Pap Smear test results.

### 4.2 Limitations

This study also had limitations similar to other studies. Since obtaining information from people about knowledge and constructs was self‐reported, their answers may have been influenced to some extent by time, environmental factors, or a large number of questions. Also, interventional studies in this field and based on HBM that focus on holding online training sessions on a web platform were very limited, which limited the possibility of discussing and interpreting the findings of the current study. The utilization of a multi‐stage sampling method is also another limitation in this study, as it potentially hinders the attainment of an accurate representation of the population.

## 5 CONCLUSION

The results of this research showed that the educational intervention based on HBM and using the virtual platform is effective in improving cognitive variables and doing Pap smear test. Therefore, the implementation of this education method in health centers is recommended due to its cost‐effectiveness for public use, lack of human resources in the health system for face‐to‐face training, and easy access. Additionally, since interventions using these methods are newer compared to face‐to‐face education and are in the early stages of development and evaluation for training effectiveness, it is necessary to repeat more studies about these new training methods.

## AUTHOR CONTRIBUTIONS

**Mahdieh Sadat Khoshnazar:** Conceptualization (equal); data curation (equal); investigation (equal); methodology (equal); project administration (equal); validation (equal); visualization (equal); writing – original draft (equal); writing – review and editing (equal). **Mohammad Javad Tarrahi:** Formal analysis (equal); methodology (equal); software (equal); writing – review and editing (equal). **Hossein Shahnazi:** Conceptualization (equal); data curation (equal); formal analysis (equal); funding acquisition (equal); investigation (equal); methodology (equal); project administration (equal); resources (equal); software (equal); supervision (equal); validation (equal); visualization (equal); writing – original draft (equal); writing – review and editing (equal).

## FUNDING INFORMATION

Research Deputy of Isfahan University of Medical Sciences partially funded this study. We declare that, the supporting source/financial relationships had no involvement in study design; collection, analysis, and interpretation of data; writing of the report; or the decision to submit the report for publication.

## CONFLICT OF INTEREST STATEMENT

The authors have stated explicitly that there are no conflicts of interest in connection with this article.

## ETHICS STATEMENT

This article is a part of the MSc thesis of Health Education, which is approved by the Research Deputy of Isfahan University of Medical Sciences.

## Data Availability

All authors have read and approved the final version of the manuscript. Corresponding author had full access to all of the data in this study and takes complete responsibility for the integrity of the data and the accuracy of the data analysis.
